# Recent Trends in Clinical Trials for Pediatric Sarcoma in the United States: An Analysis of ClinicalTrials.gov

**DOI:** 10.3390/children13040455

**Published:** 2026-03-26

**Authors:** Khaled Alkhawaldeh, Signe Thorpe, Sukjoo Cho, Alexandra Miller, Maua Alleyne, Jennifer Jones, Lynda Beaupin, Ajay Gupta, Jonathan Metts

**Affiliations:** 1 Sarcoma Department, H. Lee Moffitt Cancer Center and Research Institute, Tampa, FL 33612, USA; jonathan.metts@moffitt.org; 2 MACC Fund Center for Cancer and Blood Disorders, Children’s Wisconsin, 8915 W Connell Ct, Milwaukee, WI 53226, USA; 3 Aflac Cancer and Blood Disorders Center, Children’s Healthcare of Atlanta, Emory University, Atlanta, GA 30322, USA; sukjoo.cho@emory.edu; 4 Data Coordinating Center for Pediatric Multicenter Studies, Institute for Clinical and Translational Research, Johns Hopkins All Children’s Hospital, St. Petersburg, FL 33701, USA; 5 Cancer and Blood Disorders Institute, Johns Hopkins All Children’s Hospital, St. Petersburg, FL 33701, USA; 6 Ascentage Pharma Group Inc., Rockville, MD 20850, USA; lynda.beaupin@ascentage.com; 7 Division of Pediatric Oncology, Department of Pediatrics, Roswell Park Comprehensive Cancer Center, Jacobs School of Medicine and Biomedical Sciences, University at Buffalo, Buffalo, NY 14263, USA; ajay.gupta@roswellpark.org

**Keywords:** pediatric sarcoma, clinical trials, ClinicalTrials.gov, rare cancers, pediatric oncology

## Abstract

**Highlights:**

**What are the main findings?**

- Most sarcoma clinical trials enrolling children in the United States include both pediatric and adult patients rather than being pediatric-specific.
- Pediatric sarcoma trials are predominantly early-phase studies focused on drug or biologic therapies and are largely conducted across multiple institutions.

**What are the implications of the main findings?**

- Limited pediatric-only trials may restrict age-specific insights into treatment efficacy and toxicity for children with sarcoma.
- Expanded pediatric-focused trial designs and improved accrual strategies are needed to advance outcomes in this underserved population.

**Abstract:**

**Background/Objectives**: Pediatric sarcomas are rare and heterogeneous malignancies for which clinical trials are essential to advance treatment and improve outcomes. However, the characteristics and trends of sarcoma clinical trials enrolling children in the United States have not been comprehensively described. This study aimed to characterize U.S.-based sarcoma clinical trials enrolling pediatric patients and to evaluate trends over time. **Methods**: ClinicalTrials.gov was searched for interventional sarcoma trials conducted in the United States that enrolled patients ≤ 17 years of age and were posted between 27 September 2007 and 11 January 2023. Trials were categorized as pediatric (maximum eligible age ≤ 21 years) or pediatric/adult (>21 years). Trial characteristics, including phase, intervention type, funding source, geographic scope, and reasons for early termination, were analyzed. **Results**: A total of 273 eligible trials were identified, of which 79% enrolled both pediatric and adult patients. Most studies were early phase (Phase 1, 2, or 1/2; 59%) and primarily evaluated drug or biologic therapies (73%). Trials involving mixed cancer types were most common (26%). The majority were multi-institutional (66%), non-industry funded (57%) and conducted exclusively in the United States (75%). Trial activations increased over time (*p*-value = 0.01), with a higher proportion of industry-funded studies initiated between 2016 and 2022 (*p*-value = 0.009). Twenty-three trials (8.4%) were terminated early, most commonly due to slow accrual (39%). **Conclusions**: Most sarcoma clinical trials enrolling pediatric patients continue to include both adult and pediatric populations, which may limit the development of therapies tailored to the unique biology of pediatric sarcomas. Improving outcomes will require greater emphasis on pediatric-focused research, enhanced collaboration across institutions, and increased awareness of clinical and regulatory frameworks to support the initiation of industry-funded trials.

## 1. Introduction

Sarcomas are malignant tumors of mesenchymal origin that arise in bone, cartilage, and soft tissues [1,2]. They encompass a large number of histological subtypes, making each individual sarcoma type very rare. Advances in molecular characterization, including the identification of recurrent fusion genes, have further refined sarcoma classification, resulting in increasingly specific but rarer disease subtypes [3,4,5]. Although embryonal tumors (e.g., neuroblastoma, retinoblastoma, and hepatoblastoma) are more common in early childhood and epithelial malignancies predominate in adulthood, sarcomas are unique in that they occur across the entire age spectrum, with marked variation in histologic distribution by age group [2,6,7].

The relative burden of sarcoma also differs across pediatric, adolescent, and young adult populations.

Approximately 15% of sarcoma diagnoses occur in adolescents aged 15–19 years, whereas sarcomas account for only about 3% of all cancer diagnoses in adults aged 30–39 years [6,7,8]. Histological patterns also vary with age. Rhabdomyosarcoma is the most common soft tissue sarcoma in children aged 0–14 years [9], while osteosarcoma and Ewing sarcoma are most frequently diagnosed during adolescence [10,11]. Osteosarcoma predominates in adolescence, whereas a broader range of soft tissue sarcomas become more common in early adulthood [10,12].

Since the incorporation of chemotherapy into sarcoma treatment regimens in the 1970s, survival outcomes have improved for several sarcoma subtypes, although benefits vary by tumor biology and patient age [12]. For example, patients with Ewing sarcoma now achieve approximately 75% five-year disease-free survival with multimodal therapy that includes combination chemotherapy [13], and outcomes for osteosarcoma have similarly improved with the addition of postoperative chemotherapy [14]. In contrast, long-term survival gains in metastatic sarcoma have been more limited [15]. In pediatric sarcomas, cytotoxic chemotherapy remains the cornerstone of systemic therapy for most histologic subtypes, and few novel agents have demonstrated substantial benefit in the newly diagnosed setting to date [16].

Advancing therapy for pediatric sarcoma will therefore require the development and evaluation of new therapeutic strategies, with clinical trials serving as the primary mechanism for testing emerging treatments in this population [17]. Understanding the current landscape of clinical trials is essential to identify gaps in trial availability, barriers to accrual, and opportunities for innovation. To date, a comprehensive analysis of completed and ongoing sarcoma clinical trials enrolling pediatric patients in the United States has not been reported. The primary objective of this study was to characterize the features of sarcoma clinical trials enrolling children in the United States.

Secondary objectives included comparing trials based on age eligibility, industry sponsorship, and temporal trends in trial activation.

## 2. Materials and Methods

### 2.1. Data Source and Trial Inclusion

ClinicalTrials.gov is a United States-based web registry with information on both publicly and privately funded clinical studies, including clinical trials. Summaries of study protocols are presented, including but not limited to diseases, interventions, study design, eligibility criteria, and study locations. Trial records were queried for those with a start date between 27 September 2007, to 11 January 2023. Notably, 27 September 2007, marks the date of mandatory trial reporting in ClinicalTrials.gov by Section 801 of the Food and Drug Administration Amendments Act of 2007. This data was accessed on 11 January 2023. Trial posting dates on ClinicalTrials.gov may precede trial start dates, resulting in some trials with start dates after the date of data accession, including some trials with a listed start date in 2023. Trials starting in 2023 remained in the overall analysis but were excluded from the temporal trend analysis.

Two reviewers (A.G. and J.M.) performed independent manual reviews of all trials. Any discrepancies between these reviewers were discussed and resolved, with a third reviewer (L.B.) responsible for the final determination for any unresolved discrepancies. Trials were filtered based on the condition search term “cancer” and other search terms to identify those trials involving sarcoma-specific histologies (Table 1). Trials were included if they had a lower bound age of eligibility ≤ 17 years by using the automated age search filter, had a study type as interventional, and enrolled at least one site in the United States. Trials were excluded if they were withdrawn before enrolling any patients, did not enroll patients 17 years of age and younger, did not primarily study anti-cancer therapy, did not enroll sarcoma in general or its subtypes listed in Table 1, if the trial was found to be non-interventional, or if the trial focused only on long-term follow-up from a prior trial.

### 2.2. Study Variables

The cancer type for each trial was categorized as “sarcomas” if multiple sarcoma histologies were eligible, “solid tumor” if sarcomas were eligible in addition to other solid tumor types, and “mixed” if a sarcoma was eligible in addition to cancer types outside of solid tumors (e.g., central nervous system tumors, leukemia/lymphoma). Trial phases were categorized as phase 1, phase 1/2 or 2, phase 2/3 or 3, phase 4, or not stated. The interventions under investigation were classified as drug/biologic, procedure, radiation, stem cells/cellular therapy, or multiple interventions. We initially classified trials as “children only” based on a maximum age of ≥17; however, very few trials met this definition. Therefore, we reclassified trials as “pediatric” if they included patients ages ≤ 21 years or “pediatric/adult” if patients were ages > 21 years. Our rationale for the pediatric group was that pediatric oncology practices routinely treat patients up to 21 years of age. Sponsorship was categorized as industry, non-industry, or combined. Study sites were categorized as United States only or as international if at least one participating site was outside the United States. For “number of sites”, trials were categorized as single-institution or multiple-institution studies. For trials listed as “completed” on Clinicaltrials.gov at the time of data access, the trial duration was determined based on the reported start date and primary completion date. For studies classified as “terminated” at the time of data access, the reason for termination was categorized as COVID-19, drug availability, drug development decision, lack of effect, slow accrual, or unknown.

### 2.3. Statistical Analysis

Characteristics of interest were examined as a whole and stratified to compare (1) pediatric-only versus adult/pediatric trials and (2) trials receiving any industry funding vs. no industry funding. Continuous variables were summarized as means with standard deviations or medians with interquartile ranges (IQR) and compared by parametric or non-parametric tests according to the sample distribution. For comparisons of two independent groups, the Wilcoxon rank-sum test was used for non-normally distributed continuous data. Categorical variables were summarized as counts with percentages and compared using Chi-square tests or Fisher’s exact tests as appropriate. The Mann–Kendall trend test was used to investigate temporal trends in trial frequency. All statistical analyses were two-sided, performed using R version 4.1.2 (1 November 2021), with *p*-value < 0.05 considered statistically significant.

## 3. Results

### 3.1. Trial Search and Final Inclusion

**Figure 1:**
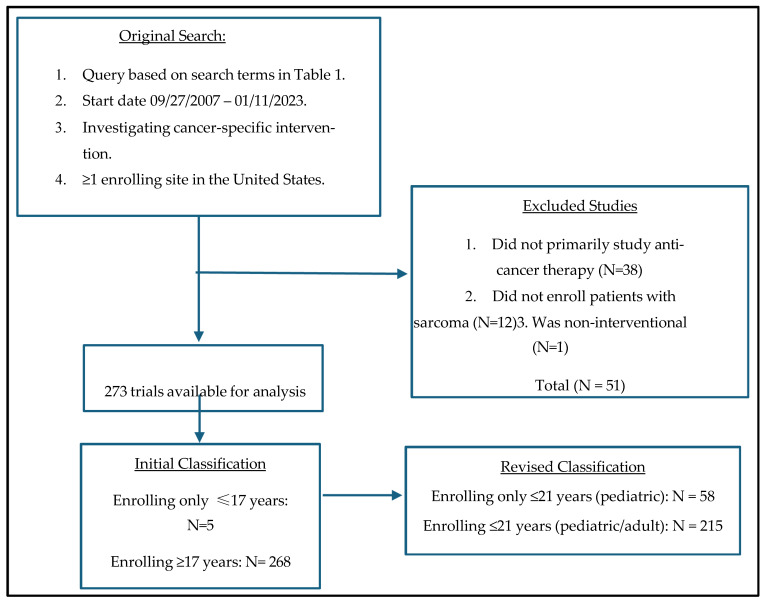
Flow chart depicting study inclusion and exclusion criteria. No final inclusion discrepancies were identified between the two reviewers.

During the primary search, 324 trials were identified. On manual review, 51 trials were excluded based on the inclusion/exclusion criteria, resulting in 273 eligible trials included in the final analysis (Figure 1).

### 3.2. Overall Characteristics of Trials

Of 273 trials identified, only 5 trials were enrolling exclusively “children only” (17 years of age and younger); all were drug/biologic studies. Therefore, our analysis focused on the two reclassified groups: pediatric (maximum age of eligibility ≤ 21 years) or pediatric/adult (maximum age of eligibility > 21 years). Based on these groupings, there were 215 (79%) pediatric/adult trials, while 58 (21%) were pediatric. Table 2 demonstrates the overall characteristics of these trials. The most common diagnosis types were mixed (*n* = 70, 26%), multiple solid tumors (*n* = 64, 23%), and multiple sarcomas (*n* = 59, 22%). Most trials were Phase 1/2 or 2 (*n* = 161, 59%), and fifteen were Phase 2/3 or 3 trials (5.5%). The most common interventional type was drug/biologic agents (*n* = 199, 73%). Notably, 31 trials (11%) involved stem cell transplant or cellular therapy, and 31 trials (11%) used multiple intervention types. The majority of trials received no industry funding (*n* = 155, 57%). Most studies were exclusive to the United States (*n* = 203, 75%), and multi-institutional (*n* = 180, 66%). Several trials were actively recruiting at the time of data accession (*n* = 97, 36%), with 83 trials (30%) completed. The median duration of completed trials was 4 years (range 0.8–9.8 years). Twenty-three trials (8.4%) were prematurely terminated, with the most common reasons being slow accrual (39%) and drug development decisions (26%).

### 3.3. Trial Characteristics Stratified by Age of Eligibility

Trial characteristics stratified by age eligibility are shown in Table 2. Pediatric trials differed significantly from pediatric/adult trials with respect to cancer type distribution (*p* < 0.001). Mixed-histology enrollment was more common in pediatric trials (62%) compared with pediatric/adult trials (16%).

No statistically significant differences were observed between the two groups with respect to trial phase, intervention type, funding source, number of participating sites, geographic location, or reasons for trial termination. Although not statistically significant, pediatric trials demonstrated a longer median duration compared with pediatric/adult trials (4.8 vs. 3.65 years; *p* = 0.08).

### 3.4. Trial Characteristics Stratified by Funding Source

Trial characteristics stratified by funding source are presented in Table 3. Trials receiving any industry funding (industry or combined sponsorship) differed significantly from non-industry-funded trials in sarcoma type distribution (*p* = 0.01), although trials enrolling multiple sarcoma histologies were the most common category in both groups.

Funding source was also associated with trial phase (*p* = 0.002). A higher proportion of non-industry-funded trials were phase 1 studies, whereas trials with any industry funding more frequently consisted of phase 1/2 or phase 2 studies (69% vs. 50%). Industry-funded trials were more likely to evaluate drug or biologic interventions (86% vs. 63%, *p* < 0.001) and to include international study sites (41% vs. 14%, *p* < 0.001). Industry-funded trials also more commonly involved multiple institutions (76% vs. 58%, *p* = 0.002).

### 3.5. Temporal Trends in Trial Activation

**Figure 2:**
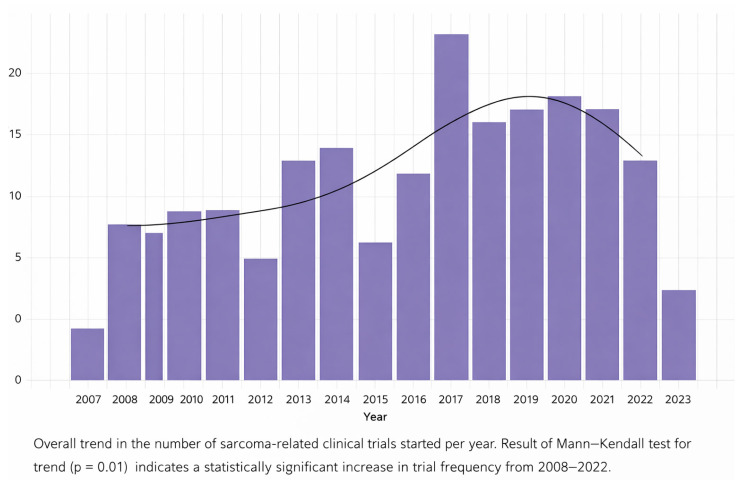
Overall trend in number of clinical trials started per year.

Annual trial activations by listed start date are shown in Figure 2. There was a significant increase in the number of sarcoma clinical trials initiated annually from 2008 through 2022 (*p* = 0.01). A median of 18 trials (range: 10–27) were initiated per year, with a peak of 27 trials in 2017.

**Figure 3:**
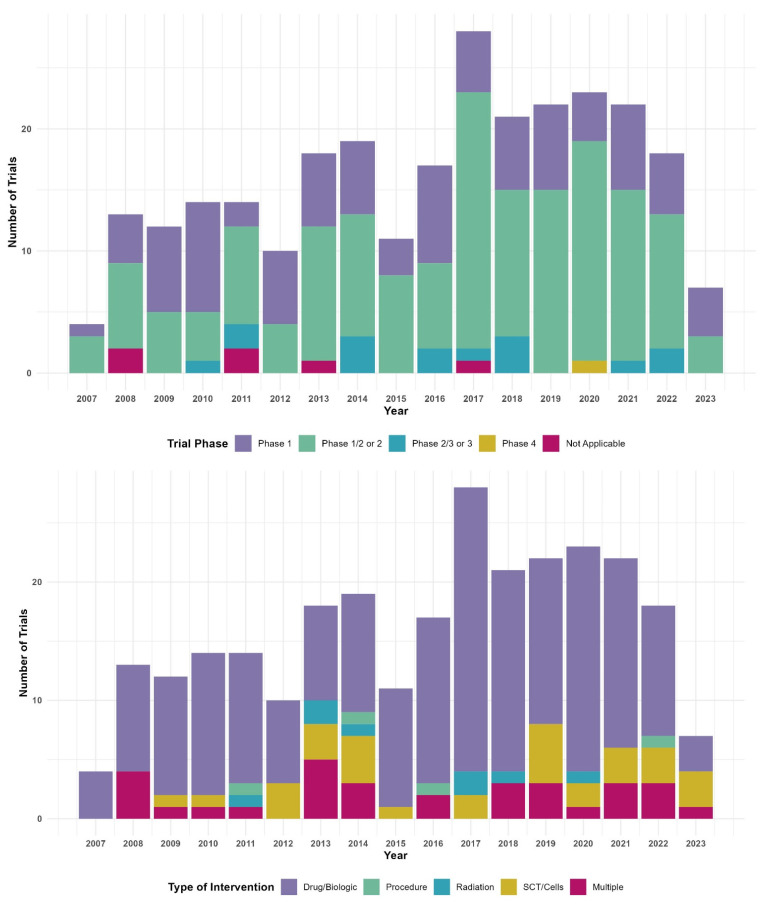

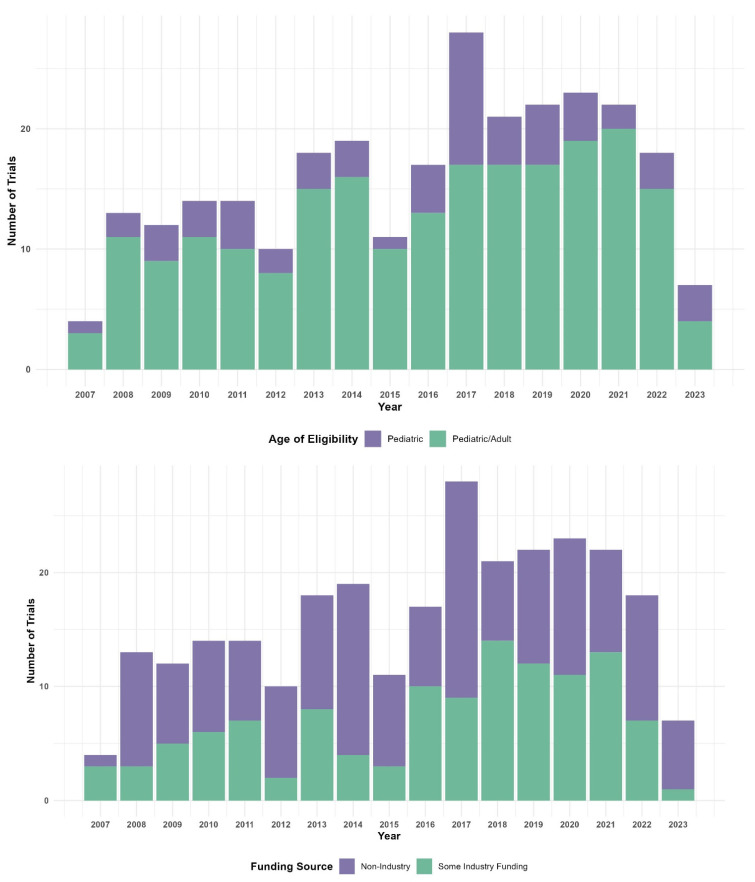
Number of clinical trials started per year by trial phase, age of eligibility, type of intervention and funding source.

Although the overall trend in the proportion of trials with industry funding over time did not reach statistical significance (*p* = 0.09), stratified analysis demonstrated that 34% of trials initiated between 2008 and 2015 had industry involvement compared with 50% of trials initiated between 2016 and 2022 (*p* = 0.009). Additional analyses examining temporal trends by trial phase, age eligibility, intervention type, and funding source are presented in Figure 3, with no consistent trends observed.

Additional analyses of temporal trends by trial phase and intervention type are provided in Figures S1 and S2, respectively.

## 4. Discussion

This study analyzes the characteristics and trends in clinical trials enrolling children with sarcoma in the United States between 2007 and 2023. Most clinical trials were designed for the pediatric/adult population. Many trials studied mixed histologies and were mainly early-phase (Phase 1, 1/2 or 2) studies. Drugs and biological therapies were the most common treatment approaches. An increase in yearly activations was noted. The most common reasons for trial closure were slow accrual and issues with drug development. Industrially funded trials were conducted more often internationally.

The study highlights important progress as well as challenges in clinical trials for sarcoma in children. It demonstrates a gradual increase in the activation of pediatric sarcoma trials enrolling children over the study period. This may reflect concerted efforts within the pediatric oncology community to develop new therapeutic options for this rare group of cancers, even though this number is still limited in comparison with other types of cancer.

Another encouraging result was the increase in industry-sponsored trials during 2016–2022 (Figure 3). A potential influential factor may be the passage of the 21st Century Cures Act, which was enacted in December 2016, which gave financial support for rare pediatric cancers and diseases to facilitate faster drug development and approval [18]. Another contributing factor may be the Research to Accelerate Cures and Equity (RACE) for Children Act, which was signed into law in 2017 and fully implemented on 17 August 2020, that requires drug developers—who intend to apply for FDA approval of certain adult cancer drugs—to include in their investigative plans how the drug will also be studied in pediatric patients as appropriate [19].

The current approach to pediatric sarcoma clinical trials often groups various histological subtypes in mixed sarcoma or mixed solid tumor trials [20]. This is likely due to early-phase studies often recruiting across histologies for purposes of dose-finding, or if different sarcoma diagnoses may benefit from a treatment [21]. However, this approach fails to examine the biological and molecular differences between tumor types [22]. The lack of histological specificity remains a challenge to producing disease-specific data for this age group [23].

In addition, the frequent inclusion of both pediatric and adult populations in sarcoma trials may further complicate the interpretation of treatment efficacy and toxicity profiles specific to pediatric patients, highlighting the need for strategies that facilitate greater enrollment in pediatric-focused clinical trials.

A potential barrier to trial completion was slow patient accrual and drug development challenges. However, pediatric-only trials reported slow accrual in only one case, making up 25% of termination reasons. Even though the sample size is small, this suggests that when pediatric-specific trials are available, they tend to attract sufficient participants. Multiple variables contribute to slow accrual, such as the number and location of participating institutions, competing trials, and the timing of trial opening. Disease-specific factors also play a role, such as the annual incidence and types of targeted cancers [24]. Drug development challenges were the second most common reason for trial termination. However, recent advancements demonstrate not just regulatory progress but also methodological rigor in pediatric approvals. For example, the IWILFIN, also known as eflornithine or DFMO (difluoromethylornithine), received Food and Drug Administration (FDA) approval for maintenance therapy in high-risk neuroblastoma following a nonrandomized, externally controlled study [25].

Clinical trials using diverse treatment approaches were identified in this study, including drug/biologic therapies, SCT/cellular therapies, multiple modalities, and other less common approaches, such as surgery and radiation. In 2024, the Food and Drug Administration (FDA) approved the first treatment for sarcoma, Tecelra^®^ (afamitresgene autoleucel), a gene therapy for treating unresectable or metastatic synovial sarcoma [26]. This represents the first gene therapy and first T-cell receptor (TCR)-engineered cellular therapy approved for the treatment of synovial sarcoma. Unfortunately, even with a trial like SPEARHEAD3 that is open to testing this drug in children, this may delay access to this medication for pediatric patients in the standard-of-care setting, despite the fact that synovial sarcoma is the most common non-rhabdomyosarcoma soft tissue sarcoma (NRSTS) histology [27].

Future studies examining specific therapeutic agents and treatment strategies investigated in sarcoma clinical trials may help further clarify evolving treatment approaches and drug development efforts in pediatric sarcoma.

In many cases, these approaches are less effective due to differences in pediatric physiology, mutations, and tumor behavior. For example, while checkpoint inhibitors have shown success in adult solid tumors, they have not demonstrated the same efficacy in pediatric patients [28].

On the other hand, cellular therapies offer a promising future for pediatric sarcomas. Therapies such as CAR T-cells, HER2-targeted treatments, and other innovative cellular approaches are currently being tested in multiple trials.

These advancements bring hope for progress in the treatment of pediatric sarcomas, potentially changing the way for significant breakthroughs in this field [29].

A notable difference related to funding sources was the higher percentage of industrially funded studies directed toward adult sarcoma trials. These trials were more likely to be international and multi-institutional in comparison to those funded by non-industry sources; this means there is a more attractive environment for these industrially funded trials internationally. Further investigation is needed to better understand these differences and to identify strategies that may facilitate the development of additional pediatric sarcoma trials in the future. Additionally, there has been a gradual increase in industrially funded studies over time, reflecting broader reach and resources typically available in commercially sponsored research.

### Limitations

The study faced limitations as it was built on data from ClinicalTrials.gov. First, smaller trials or those not meeting criteria are often underreported [30]. Second, we used specific keywords and age groups to collect data on pediatric sarcoma, but it is possible that all sarcoma trials enrolling children were not captured. Third, the FDA Amendments Act (FDAAA) of 2007 requires the reporting of results for most FDA-regulated trials, but it provides exemptions for certain studies, such as observational trials or early-phase trials, which may then be missing from our data collection [31].

Future studies comparing sarcoma clinical trials enrolling pediatric patients with trials restricted to adult populations may provide additional insight into differences in trial availability, design, and industry sponsorship across age groups.

## 5. Conclusions

This analysis highlights progress and ongoing challenges in pediatric sarcoma clinical trials since 2007. While an increase in trial activation indicates the scientific and medical community’s drive to find treatment for these rare groups of cancers, the significant gap in pediatric-specific trials remains a critical issue. Most trials continue to include both adults and children, limiting the development of tailored therapies that address the unique biology of pediatric sarcomas. To improve outcomes, there is an urgent need for pediatric-focused research, enhanced collaboration across institutions, and increased awareness of clinical and governmental regulations to facilitate the initiation of industry-funded trials.

## Figures and Tables

**Table 1:** Additional search terms were used to identify clinical trials investigating sarcoma that enrolled pediatric patients.

Sarcoma
Bone Sarcoma
Soft Tissue Sarcoma Osteosarcoma
Rhabdomyosarcoma
Non-rhabdomyosarcoma soft tissue tumor (NRSTS)
Soft Tissue Tumor (STS)
Ewing Sarcoma/Ewing/Ewings/Ewing’s

**Table 2:** Characteristics of sarcoma clinical trials enrolling children, stratified by age of eligibility. Abbreviations: AS: angiosarcoma, ASPS: alveolar soft part sarcoma, CCS: clear cell sarcoma, DSRCT: desmoplastic small round cell tumor, ES: Ewing Sarcoma, LMS: leiomyosarcoma, LPS: liposarcoma, MPNST: malignant peripheral nerve sheath tumor, OS: osteosarcoma, RMS: rhabdomyosarcoma, SS: synovial sarcoma, US: United States, IQR: interquartile range, ST: Soft Tissue.

Age of Eligibility					
Category	Variable	Overall, N = 273 ^1^	Pediatric, N = 58 ^1^	Pediatric/Adult, N = 215 ^1^	*p*-Value ^2^
Cancer Type	AS	1 (0.4%)	0 (0%)	1 (0.5%)	<0.001
	ASPS	3 (1.1%)	1 (1.7%)	2 (0.9%)	
	CCS	1 (0.4%)	0 (0%)	1 (0.5%)	
	DSRCT	2 (0.7%)	0 (0%)	2 (0.9%)	
	ES	19 (7.0%)	0 (0%)	19 (8.8%)	
	LMS	1 (0.4%)	0 (0%)	1 (0.5%)	
	LPS	2 (0.7%)	0 (0%)	2 (0.9%)	
	MPNST	4 (1.5%)	0 (0%)	4 (1.9%)	
	OS	32 (12%)	1 (1.7%)	31 (14%)	
	RMS	10 (3.7%)	2 (3.4%)	8 (3.7%)	
	Sarcomas	59 (22%)	4 (6.9%)	55 (26%)	
	SS	5 (1.8%)	0 (0%)	5 (2.3%)	
	ST	64 (23%)	14 (24%)	50 (23%)	
	Mixed	70 (26%)	36 (62%)	34 (16%)	
Trial Phase	Phase 1	90 (33%)	21 (36%)	69 (32%)	0.6
	Phase 1/2 or 2	161 (59%)	35 (60%)	126 (59%)	
	Phase 2/3 or 3	15 (5.5%)	1 (1.7%)	14 (6.5%)	
	Phase 4	1 (0.4%)	0 (0%)	1 (0.5%)	
	Not Stated	6 (2.2%)	1 (1.7%)	5 (2.3%)	
Intervention Type	Drug/Biologic	199 (73%)	48 (83%)	151 (70%)	0.3
	Procedure	4 (1.5%)	0 (0%)	4 (1.9%)	
	Radiation	8 (2.9%)	2 (3.4%)	6 (2.8%)	
	Stem Cells/Cell Therapy	31 (11%)	5 (8.6%)	26 (12%)	
	Multiple	31 (11%)	3 (5.2%)	28 (13%)	
Funding Source	Industry	68 (25%)	13 (22%)	55 (26%)	0.4
	Non-Industry	155 (57%)	37 (64%)	118 (55%)	
	Combination	50 (18%)	8 (14%)	42 (20%)	
Study Site	US only	203 (75%)	42 (72%)	161 (75%)	0.7
	International	69 (25%)	16 (28%)	53 (25%)	
	Unknown	1	0	1	
Number of Sites	Single	93 (34%)	17 (29%)	76 (35%)	0.4
	Multiple	180 (66%)	41 (71%)	139 (65%)	
Study Status	Recruiting	97 (36%)	19 (33%)	78 (36%)	0.8
	Enrolling by invitation	1 (0.4%)	0 (0%)	1 (0.5%)	
	Not yet recruiting	10 (3.7%)	4 (6.9%)	6 (2.8%)	
	Completed	83 (30%)	19 (33%)	64 (30%)	
	Active, not recruiting	53 (19%)	11 (19%)	42 (20%)	
	Terminated	23 (8.4%)	4 (6.9%)	19 (8.8%)	
	Suspended	4 (1.5%)	1 (1.7%)	3 (1.4%)	
	Unknown status	2 (0.7%)	0 (0%)	2 (0.9%)	
Reason for Termination	N	23	4	19	0.2
	COVID-19	1 (4.3%)	1 (25%)	0 (0%)	
	Drug Availability	2 (8.7%)	0 (0%)	2 (11%)	
	Drug Development Decision	6 (26%)	1 (25%)	5 (26%)	
	Lack of Effect	2 (8.7%)	0 (0%)	2 (11%)	
	Lack of Funding	1 (4.3%)	1 (25%)	0 (0%)	
	Slow Accrual	9 (39%)	1 (25%)	8 (42%)	
	No Reason Provided	2 (8.7%)	0 (0%)	2 (11%)	
Duration of Trial	N	83	19	64	0.08
(years, completed only)	Median (IQR)	4.00 (2.50, 5.25)	4.80 (3.75, 5.70)	3.65 (2.40, 4.73)	
	Range	0.80, 9.80	0.90, 9.80	0.80, 9.00	

^1^ Values are presented as a number (percentage). ^2^ *p*-values were calculated using the chi-square test.

**Table 3:** Characteristics of sarcoma clinical trials enrolling children by funding source. Abbreviations: AS: angiosarcoma, ASPS: alveolar soft part sarcoma, CCS: clear cell sarcoma, DSRCT: desmoplastic small round cell tumor, ES: Ewing sarcoma, LMS: leiomyosarcoma, LPS: liposarcoma, MPNST: malignant peripheral nerve sheath tumor, OS: osteosarcoma, RMS: rhabdomyosarcoma, SS: synovial sarcoma, US: United States, IQR: interquartile range, ST: Soft Tissue.

Category	Variable	Overall N = 273 ^1^	Non-Industry N = 155 ^1^	Any Industry Funding N = 118 ^1^	*p*-Value ^2^
Sarcoma Type	AS	1 (0.4%)	0 (0%)	1 (0.8%)	0.01
	ASPS	3 (1.1%)	3 (1.9%)	0 (0%)	
	CCS	1 (0.4%)	0 (0%)	1 (0.8%)	
	DSRCT	2 (0.7%)	0 (0%)	2 (1.7%)	
	ES	19 (7.0%)	9 (5.8%)	10 (8.5%)	
	LMS	1 (0.4%)	0 (0%)	1 (0.8%)	
	LPS	2 (0.7%)	0 (0%)	2 (1.7%)	
	MPNST	4 (1.5%)	0 (0%)	4 (3.4%)	
	OS	32 (12%)	18 (12%)	14 (12%)	
	RMS	10 (3.7%)	9 (5.8%)	1 (0.8%)	
	Sarcomas	59 (22%)	29 (19%)	30 (25%)	
	SS	5 (1.8%)	2 (1.3%)	3 (2.5%)	
	ST	64 (23%)	39 (25%)	25 (21%)	
	Mixed	70 (26%)	46 (30%)	24 (20%)	
Trial Phase	Phase 1	90 (33%)	62 (40%)	28 (24%)	0.002
	Phase 1/2 or 2	161 (59%)	79 (51%)	82 (69%)	
	Phase 2/3 or 3	15 (5.5%)	8 (5.2%)	7 (5.9%)	
	Phase 4	1 (0.4%)	0 (0%)	1 (0.8%)	
	Not Applicable	6 (2.2%)	6 (3.9%)	0 (0%)	
Intervention Type	Drug/Biologic	199 (73%)	97 (63%)	102 (86%)	<0.001
	Procedure	4 (1.5%)	4 (2.6%)	0 (0%)	
	Radiation	8 (2.9%)	6 (3.9%)	2 (1.7%)	
	SCT/Cells	31 (11%)	24 (15%)	7 (5.9%)	
	Multiple	31 (11%)	24 (15%)	7 (5.9%)	
Age of Eligibility	Pediatric	58 (21%)	37 (24%)	21 (18%)	0.2
	Pediatric/Adult	215 (79%)	118 (76%)	97 (82%)	
Study Site	US	203 (75%)	134 (86%)	69 (59%)	<0.001
	International	69 (25%)	21 (14%)	48 (41%)	
	Missing	1	0	1	
Number of Sites	Single	93 (34%)	65 (42%)	28 (24%)	0.002
	Multiple	180 (66%)	90 (58%)	90 (76%)	
Study Status	Recruiting	97 (36%)	54 (35%)	43 (36%)	0.041
	Enrolling by invitation	1 (0.4%)	0 (0%)	1 (0.8%)	
	Not yet recruiting	10 (3.7%)	8 (5.2%)	2 (1.7%)	
	Completed	83 (30%)	42 (27%)	41 (35%)	
	Active, not recruiting	53 (19%)	38 (25%)	15 (13%)	
	Terminated	23 (8.4%)	9 (5.8%)	14 (12%)	
	Suspended	4 (1.5%)	3 (1.9%)	1 (0.8%)	
	Unknown status	2 (0.7%)	1 (0.6%)	1 (0.8%)	
Reason for Termination	COVID-19	1 (4.3%)	1 (11%)	0 (0%)	0.5
	Drug Availability	2 (8.7%)	1 (11%)	1 (7.1%)	
	Drug Development Decision	6 (26%)	1 (11%)	5 (36%)	
	Lack of Effect	2 (8.7%)	1 (11%)	1 (7.1%)	
	Lack of Funding	1 (4.3%)	1 (11%)	0 (0%)	
	Slow Accrual	9 (39%)	4 (44%)	5 (36%)	
	No Reason Provided	2 (8.7%)	0 (0%)	2 (14%)	
Duration of Trial	N	83	42	41	0.9
(years, completed only)	Median (IQR)	4.00 (2.50, 5.25)	3.80 (2.40, 5.25)	4.20 (2.70, 5.20)	
	Range	0.80, 9.80	0.80, 8.00	1.00, 9.80	

^1^ Values are presented as a number (percentage). ^2^ *p*-values were calculated using the chi-square test.

## Data Availability

The data presented in this study are publicly available from ClinicalTrials.gov (https://clinicaltrials.gov) (accessed on 10 March 2023). The datasets analyzed during the current study are available from the corresponding author upon reasonable request.

## Supplementary Materials

The following supporting information can be downloaded at: https://www.mdpi.com/article/10.3390/children13040455/s1, Figure S1: Clinical trials by year and Phase; Figure S2: Clinical Trials by Year and Intervention.

## Abbreviations

APC	Article Processing Charge
AS	Angiosarcoma
ASPS	Alveolar Soft Part Sarcoma
CAR	Chimeric Antigen Receptor
CCS	Clear Cell Sarcoma
CNS	Central Nervous System
DFMO	Difluoromethylornithine
DSRCT	Desmoplastic Small Round Cell Tumor
ES	Ewing Sarcoma
FDA	Food and Drug Administration
FDAAA	Food and Drug Administration Amendments Act
HER2	Human Epidermal Growth Factor Receptor 2
IQR	Interquartile Range
IRB	Institutional Review Board
IWILFIN	Eflornithine (Difluoromethylornithine)
LMS	Leiomyosarcoma
LPS	Liposarcoma
MPNST	Malignant Peripheral Nerve Sheath Tumor
NRSTS	Non-Rhabdomyosarcoma Soft Tissue Sarcoma
OS	Osteosarcoma
RACE	Research to Accelerate Cures and Equity
RMS	Rhabdomyosarcoma
SCT	Stem Cell Transplant
SS	Synovial Sarcoma ST—Solid Tumor
US	United States

## References

[1] Bleloch J.S., Ballim R.D., Kimani S., Parkes J., Panieri E., Willmer T., Prince S. (2017). Managing sarcoma: Where have we come from and where are we going?. Ther. Adv. Med. Oncol..

[2] Arndt C.A.S., Rose P.S., Folpe A.L., Laack N.N. (2012). Common musculoskeletal tumors of childhood and adolescence. Mayo Clin. Proc..

[3] Burningham Z., Hashibe M., Spector L., Schiffman J.D. (2012). The epidemiology of sarcoma. Clin. Sarcoma Res..

[4] Casali P.G. (2015). Adjuvant chemotherapy for soft tissue sarcoma. ASCO Educ. Book.

[5] Mertens F., Antonescu C.R., Mitelman F. (2016). Gene fusions in soft tissue tumors: Recurrent and overlapping pathogenetic themes. Genes Chromosomes Cancer.

[6] Burke M.E., Albritton K., Marina N. (2007). Challenges in the recruitment of adolescents and young adults to cancer clinical trials. Cancer.

[7] Ward Z.J., Yeh J.M., Bhakta N., Frazier A.L., Girardi F., Atun R. (2019). Global childhood cancer survival estimates and priority-setting: A simulation-based analysis. Lancet Oncol..

[8] Bhatia S., Pappo A.S., Acquazzino M., Allen-Rhoades W.A., Barnett M., Borinstein S.C., Casey R., Choo S., Chugh R., Dinner S. (2023). Adolescent and young adult oncology, version 2.2024, NCCN clinical practice guidelines in oncology. J. Natl. Compr. Cancer Netw..

[9] Pappo A.S. (1996). Rhabdomyosarcoma and other soft tissue sarcomas in children. Curr. Opin. Oncol..

[10] Ottaviani G., Jaffe N. (2009). The epidemiology of osteosarcoma. Pediatric and Adolescent Osteosarcoma.

[11] Gaspar N., Hawkins D.S., Dirksen U., Lewis I.J., Ferrari S., Le Deley M.C., Kovar H., Grimer R., Whelan J., Claude L. (2015). Ewing sarcoma: Current management and future approaches through collaboration. J. Clin. Oncol..

[12] Miller K.D., Nogueira L., Devasia T., Mariotto A.B., Yabroff K.R., Jemal A., Kramer J., Siegel R.L. (2022). Cancer treatment and survivorship statistics, 2022. CA Cancer J. Clin..

[13] Tateo V., Marchese P.V., Mollica V., Massari F., Kurzrock R., Adashek J.J. (2023). Agnostic approvals in oncology: Getting the right drug to the right patient with the right genomics. Pharmaceuticals.

[14] Carbonnaux M., Brahmi M., Schiffler C., Meeus P., Sunyach M.P., Bouhamama A., Karanian M., Tirode F., Pissaloux D., Vaz G. (2019). Very long-term survivors among patients with metastatic soft tissue sarcoma. Cancer Med..

[15] Subbiah V. (2015). Fast-tracking novel drugs in pediatric oncology. Cell Cycle.

[16] Pan M., Zhou M., Xie L., Bui N., Ganjoo K. (2024). Recent advances in sarcoma therapy: New agents, strategies and predictive biomarkers. J. Hematol. Oncol..

[17] Gabay M. (2017). 21st century cures act. Hosp. Pharm..

[18] Zettler M.E. (2022). The RACE for children act at one year: Progress in pediatric development of molecularly targeted oncology drugs. Expert Rev. Anticancer Ther..

[19] Lee D.Y., Staddon A.P., Shabason J.E., Sebro R. (2019). Phase I and phase II clinical trials in sarcoma: Implications for drug discovery and development. Cancer Med..

[20] Carmagnani Pestana R., Moyers J.T., Roszik J., Sen S., Hong D.S., Naing A., Herzog C.E., Fu S., Piha-Paul S.A., Rodon J. (2023). Impact of biomarker-matched therapies on outcomes in patients with sarcoma enrolled in early-phase clinical trials (SAMBA 101). Clin. Cancer Res..

[21] Nakano K., Takahashi S. (2020). Precision medicine in soft tissue sarcoma treatment. Cancers.

[22] Hingorani P., Janeway K., Crompton B.D., Kadoch C., Mackall C.L., Khan J., Shern J.F., Schiffman J., Mirabello L., Savage S.A. (2016). Current state of pediatric sarcoma biology and opportunities for future discovery. Cancer Genet..

[23] Hauck C.L., Kelechi T.J., Cartmell K.B., Mueller M. (2021). Trial-level factors affecting accrual and completion of oncology clinical trials: A systematic review. Contemp. Clin. Trials Commun..

[24] Shakeel A., Baloch A., Kumari V., Kazmi S.K.Z., Aftab K., Abid S., Syed A.M., Yousuf J.M., Hasanain M.M., Anjum M.U.M. (2024). Iwilfin (eflornithine) approved by the FDA as the first and only oral maintenance therapy for high-risk neuroblastoma in adult and pediatric patients. Medicine.

[25] Keam S.J. (2024). Afamitresgene autoleucel: First approval. Mol. Diagn. Ther..

[26] Stacchiotti S., Van Tine B.A. (2018). Synovial sarcoma: Current concepts and future perspectives. J. Clin. Oncol..

[27] Ciurej A., Lewis E., Gupte A., Al-Antary E. (2023). Checkpoint immunotherapy in pediatric oncology: Will we say checkmate soon?. Vaccines.

[28] Terry R.L., Meyran D., Fleuren E.D.G., Mayoh C., Zhu J., Omer N., Ziegler D.S., Haber M., Darcy P.K., Trapani J.A. (2021). Chimeric antigen receptor T cell therapy and the immunosuppressive tumor microenvironment in pediatric sarcoma. Cancers.

[29] Zarin D.A., Tse T., Williams R.J., Califf R.M., Ide N.C. (2011). The ClinicalTrials.gov results database—Update and key issues. N. Engl. J. Med..

[30] Von Eschenbach A.C. (2008). The FDA Amendments Act: Reauthorization of the FDA. Food Drug Law J..

[31] Anderson M.L., Chiswell K., Peterson E.D., Tasneem A., Topping J., Califf R.M. (2015). Compliance with results reporting at ClinicalTrials.gov. N. Engl. J. Med..

