# Supplementary material for: Recent Trends in Clinical Trials for Pediatric Sarcoma in the United States: An Analysis of ClinicalTrials.gov
Source: Children (Basel). 2026 Mar 26;13(4):455. doi: 10.3390/children13040455 (PMC13114444; doi:10.3390/children13040455)
Supplement: Supplementary file 1 [file children-13-00455-s001.zip › children-4203202-supplementary.pptx]

## Slide 1
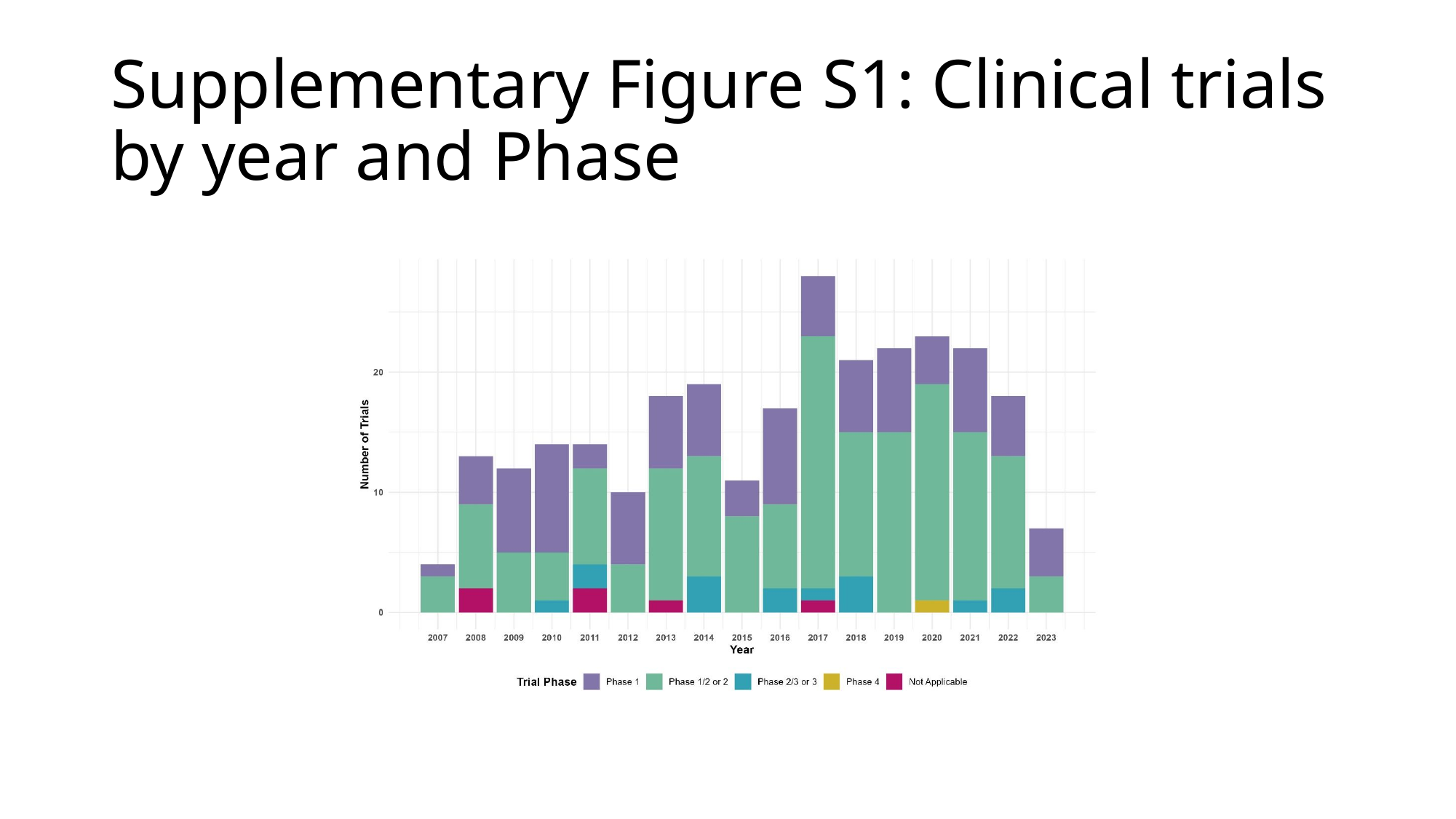

# Supplementary Figure S1: Clinical trials by year and Phase

## Slide 2
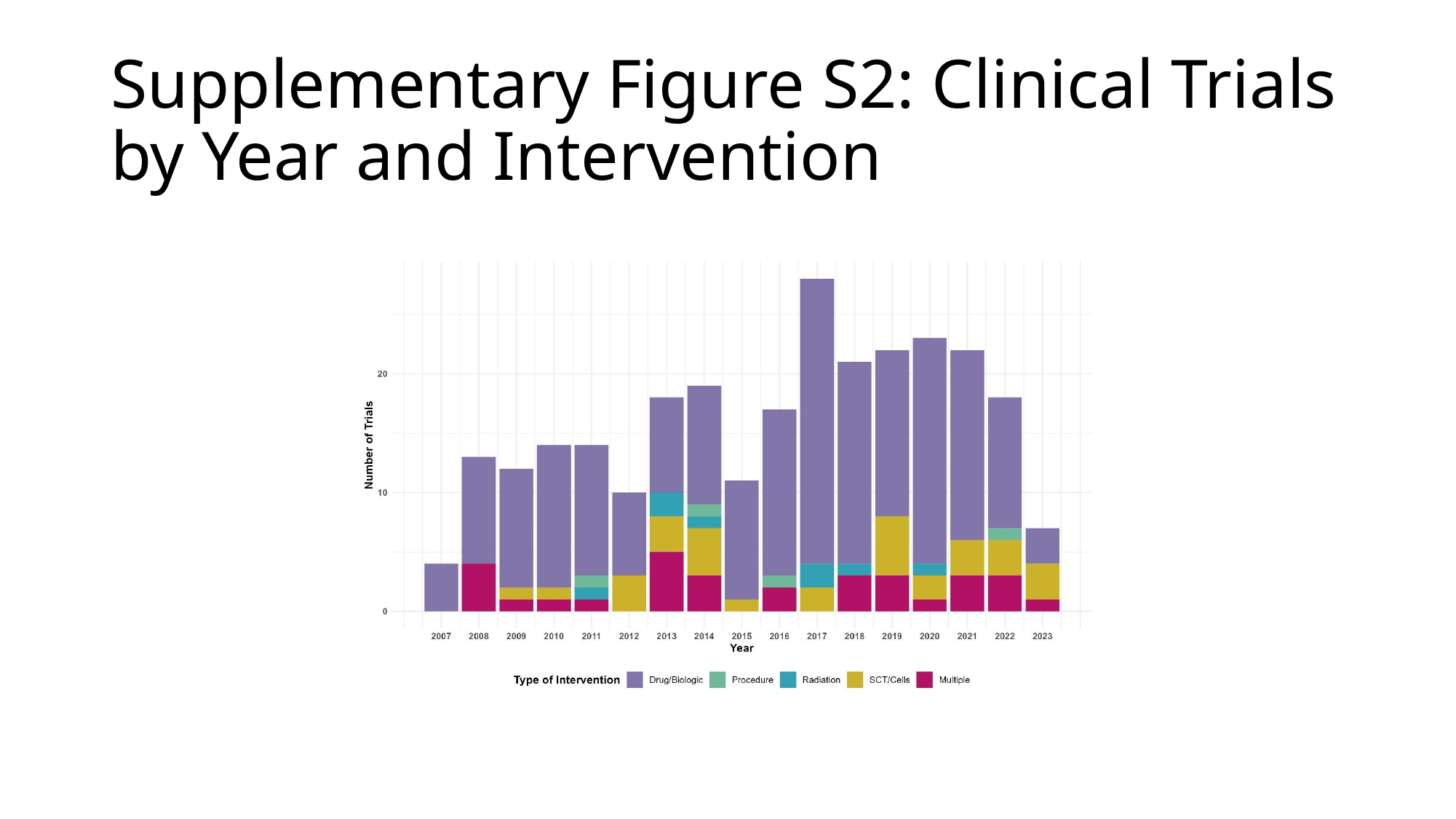

# Supplementary Figure S2: Clinical Trials by Year and Intervention
